# Psychopathic Hospitals*Read at the State Conference of Charities and Corrections in annual convention, November, 1914.

**Published:** 1915-01

**Authors:** F. S. White


### Psychopathic Hospitals.*

BY F. S. WHITE.

A psychopathic hospital is one devoted exclusively to the treatment of the psychoses. Such a hospital differs very materially from an ordinary hospital in which cases of sickness and surgical cases are treated, owing to the fact that the type of diseases treated is very different.

In an ordinary hospital it is the body which shows the effect of disease, and to its restoration to the normal conditions the efforts of the physician is directed. In a psychopathic hospital while much attention is given to the body, yet it is the mental condition which is the distinctive feature of the case, and the efforts of the physician are directed to the restoration of the mind to its normal condition. The proper location for a State psychopathic hospital is in connection with the State hospitals for the insane. The building, however, should be entirely separate from the ward buildings, and, of course, should be well ventilated and well lighted, fireproof and sanitary in every respect. Ample means of amusement and diversion for the patients should be provided, such as

*Read at the State Conference of Charities and Corrections in annual convention, November, 1914.

music, games, reading and suitable industrial occupations. Ample grounds should be provided for outdoor exercises as well as some kind of work for all cases that employment would benefit.

At the Southwestern Insane Asylum there are two psychopathic hospitals, one for men and one for women. The one for men has one hundred beds and the one for women can accommodate one hundred and twenty-five cases. The hospital for women has just recently been occupied and is proving a great aid in the treatment of acute cases of insanity.

Believing that the members of ths association are deeply interested in the welfare of the insane of Texas, I desire to present to you a short account of the management, care and treatment of the insane at the Southwestern Insane Asylum.

Since insanity has been accepted as a very curable disease, the recovery rate has been very materially increased in all hospitals for the insane. The medical profession can aid very materially in the treatment and cure of insanity by impressing the fact upon their patients of this class that their disease is not hopeless but that when treated early and properly 75 to 80 per cent are curable. The principles of rational, scientific treatment can be applied to insanity just as to any other disease.

There is no sovereign specific for insanity, nor is there for pneumonia or typhoid fever. The very nature of the disease makes it necessary that practically all cases of insanity should be placed in institutions where special arrangements exist for their care and treatment.

When a case is admitted into the Southwestern Insane Asylum, an attempt is made to secure a complete history of the case. The family history is carefully investigated as far back as is possible. Inquiry is made into the peculiarities and habits of his ancestors, especially with reference to any warps or twists in their nervous or mental conditions. That which in one generation was considered as only some trivial mental peculiarity which could scarcely be classed as a departure from the normal may show in the next generation, especially if accentuated by peculiarities in both parents, epilepsy, insanity, imbecility or idiocy will be the result.

In securing the history, an attempt is made to determine what effect the use of narcotics and stimulants used by the parents have had on the offspring. The individual history of the case is then sought in all its details as to habits, education, environment, religion, mode of living and in fact everything possible that can be learned from the cradle to the grave.

It is with the greatest difficulty that a complete and satisfactory history can be secured. Usually ignorance is the great obstacle in the way, but often a foolish pride prevents relatives from uncovering a mental, moral or physical delinquency in the family.

Upon admission, every case is given a thorough physical examination, which is made a matter of record. The urine is given a chemical and microscopical examination. A complete blood examination is made, and in certain cases the blood pressure is taken—when indicated, a spinal puncture is made. The patient is put to bed and kept there for at least one week, during which time close observations are made. At the end of the week in bed we are better prepared to intelligently enter upon a line of treatment which is directed first to the correction of any physical ailment that may exist and to the establishment of the normal functions of all the organs.

As most acute cases before admission have been given large quantities of narcotics and sedatives, an eliminative line of treatment is indicated. We now begin on an upbuilding course, which consists of tonics and food. As in all other diseases, so in the psychoses nourishment is our sheet anchor. There is no disease that exhausts so rapidly the vital forces as an acute case of insanity. These cases are fed every ounce of food that they can assimilate. If they take food voluntarily, so much the better; if not, they are forcibly fed. They must get nourishment, and they get it when necessary from three to six times a day.

We treat acute cases of insanity upon the principles that they are acutely ill and that their strength and vital forces must be conserved, and nothing is left undone that is believed will aid in the consumption of this happy result.

With this line of treatment diligently and systematically followed, it is very seldom that it is necessary to resort to sedatives or narcotics. Prolonged warm baths will often produce refreshing sleep when hypnotics would be harmful. As all sedatives and narcotics lower and weaken the vital forces and reduce resistance to disease, they are avoided as far as possible. Of course, there are extreme cases where resort must be had to some form of hypnotic, not so much for the benefit of the individual as for the protection of others. To my mind, such remedies are as a rule of very doubtful efficiency.

The great desideration in the treatment of insanity is diversion. The line of thought and action must be changed. We must get these people out of themselves—do away with introspection, give them some new stimulus to a different line of thought, something to direct their mental processes into different and more healthful channels. This can be done very efficiently in many cases by bringing into our efforts the principles of psycho-therapy. Few of us realize what a power one mind has over another, or what influence the mind has over the body. Suggestion is a very efficient and influential means of the treatment of all diseases, and most especially of nervous and mental diseases. The physician who by his very manner and his approach to his patients can infuse into them the greatest amount of hope and good feeling is the one who is the most successful in the treatment of diseases of all kinds. It is an everyday occurrence for sick people to say that they always feel better when the doctor comes, and we know that this is only the result of the psychical influence often unconsciously exerted. Appreciating the value of these great principles, it is my earnest endeavor to give my patients every possible diversion. I insist on everybody connected with them keeping the corners of their mouths turned up instead of down. I will not inflict upon these unfortunate people anybody who has a grouch and who can see no pleasure in life.

The patients are given a dance once a week, and under the influence of lively music they for a time at least forget "The rooted sorrow and are enabled to raze out the written trouble of the brain and cleanse the stuffled bosom of that perilous stuff that weighs upon the heart."

Once a week I have a moving picture show, and endeavor to secure reels that have action and depict the comedies of life. At these entertainments there is an attendance of six or seven hundred, many of whom are deeply interested and display as much interést and amusement as will be shown by any ordinary audience. I also have a splendid baseball team which plays once or twice a week, and they play to an enthusiastic crowd of fans.

I have what I call my convalescent squads—one of men and one of women. These are placed in charge of my best attendants and are sent out over the country with no certain definite place to go. The object is to give them new scenery and new lines of thought which aid very materially in restoring their diseased minds to health.

Work and play, when properly directed, is on of the most important therapeutic agents at our command in the treatment of the mind diseased. Carrying out this idea, a large part of the patients are employed in some useful work. The farm, the garden, the dairy, the laundry, the sewing room and ward work all furnish means of employment.

The larger proportion of the patients come from the laboring classes, and were they closely confined, with nothing to relieve the monotony of white walls and polished floors, very few would be restored. I believe strongly in the treatment of the insane. By treatment, I do not mean simply medicine, but all the means to which I have so briefly referred.

Unfortunately, the belief is very prevalent that nothing can be done for the insane to aid in their restoration, but I tell you that the more intelligent effort that is used in the treatment of these unfortunate people the larger will be the per cent of recoveries.

Dr. W. W. Goddard, who was a number of years Superintendent of the Government Hospital for the Insane, at Washington, once said: "That it were better to have a few tombstones marking the efforts to cure these cases than to sit idly by and do nothing."

It is often stated by some men, who should be better informed, that the methods in vogue in Texas for taking care of the insane are antiquated and are far behind the most approved methods—which critics would make you believe are pursued by all of the other States.

The care and treatment of the insane in Texas is far from ideal, but not many States can boast of better methods. It is not likely that the time will ever come when not an insane person will be in jail in Texas, but, when the buildings now under construction are completed, practically all the insane will be provided for.

The results obtained in any line of endeavor determines the success or failure of the work. The results of the work done in hospitals for the insane may be estimated by four things: First in importance is the recovery rate; second is the comfort and happiness of the incurable in chronic cases; third is the death rate, and fourth is the cost of maintenance.

Now, in order to determine what is being accomplished in Texas, I have gathered information on the above mentioned points from seventy asylums in thirty-three States.

I have constructed a table which shows the recovery rate, the death rate and the per capita cost in the separate asylums and also the average of these points in each State. I will not take your time to read all, but will take only a few of the leading States:

AVERAGE RECOVERY RATE, DEATH RATE AND PER CAPITA COST.

| | Number Hospitals. | Recovery Rate. | Death Rate. | Per Capita Cost. |
|---|---|---|---|---|
| Arizona | 1 | 31.19 | 8.00 | $370 92 |
| Arkansas | 1 | 44.00 | 6.84 | 130 00 |
| California | 5 | 26.03 | 5.89 | 175 63 |
| Connecticut | 2 | 22.57 | 8.46 | 184 96 |
| Delaware | 1 | 23.71 | 12.00 | 157 04 |
| Florida | 1 | 20.09 | 9.84 | 197 40 |
| Georgia | 1 | 6.30 | 11.75 | 132 47 |
| Illinois | 6 | 26.11 | 6.28 | 157 64 |
| Indiana | 3 | 23.04 | 6.74 | 192 65 |
| Iowa | 1 | 31.00 | 6.00 | 197 00 |
| Kentucky | 3 | 24.84 | 8.45 | 151 94 |
| Kansas | 2 | 39.11 | 10.00 | 164 34 |
| Louisiana | 1 | 14.00 | 9.80 | 121 32 |
| Maine | 1 | 18.60 | 10.70 | 192 80 |
| Massachusetts | 5 | 14.05 | 8.42 | 235 84 |
| Maryland | 1 | 24.00 | 4.70 | 203 00 |
| Michigan | 2 | 27.10 | 5.64 | 179 77 |
| Minnesota | 4 | 23.20 | 9.00 | 123 97 |
| Mississippi | 1 | 24.00 | 6.30 | 150 00 |
| Missouri | 4 | 25.40 | 8.44 | 167 38 |
| Nebraska | 1 | 21.30 | 9.00 | 232 00 |
| Nevada | 1 | 47.00 | 16.66 | 193 00 |
| New Mexico | 1 | 26.10 | 12.95 | 186 97 |
| New York | 10 | 30.90 | 5.96 | 201 12 |
| Ohio | 5 | 26.92 | 7.38 | 153 99 |
| Oregon | 1 | 66.00 | 10.25 | 164 52 |
| Pennsylvania | 2 | 24.43 | 6.65 | 215 40 |
| Tennessee | 2 | 15.16 | 8.75 | 142 50 |
| Utah | 1 | 33.00 | 7.60 | 146 00 |
| Virginia | 2 | 13.91 | 7.77 | 132 97 |
| Washington | 1 | 31.92 | 8.05 | 170 55 |
| Wyoming | 1 | 44.00 | 4.80 | 188 51 |
| | 74 | 26.84 | 8.70 | $180 11 |
| Texas | 3 | 40.80 | 5.77 | $164 89 |
| Southwestern Insane Asylum | — | 50.00 | 7.22 | 159 27 |

These figures serve to show you that the asylums of Texas are not so far behind, but, on the other hand, are abreast of the times, as is shown by the results.

